# The Most Widely Disseminated COVID-19-Related Scientific Publications in Online Media: A Bibliometric Analysis of the Top 100 Articles with the Highest Altmetric Attention Scores

**DOI:** 10.3390/healthcare9020239

**Published:** 2021-02-23

**Authors:** Ji Yoon Moon, Dae Young Yoon, Ji Hyun Hong, Kyoung Ja Lim, Sora Baek, Young Lan Seo, Eun Joo Yun

**Affiliations:** Department of Radiology, Kangdong Sacred Heart Hospital, College of Medicine, Hallym University, 150, Seongan-ro, Gangdong-gu, Seoul 134-701, Korea; thanks1029@hanmail.net (J.Y.M.); whitehong2@gmail.com (J.H.H.); cosmos95@kdh.or.kr (K.J.L.); sorapig@hanmail.net (S.B.); ylseomd@naver.com (Y.L.S.); yeunjookr@naver.com (E.J.Y.)

**Keywords:** COVID-19, coronavirus, altmetrics, social media, bibliometric analysis

## Abstract

The novel coronavirus disease 2019 (COVID-19) is a global pandemic. This study’s aim was to identify and characterize the top 100 COVID-19-related scientific publications, which had received the highest Altmetric Attention Scores (AASs). Hence, we searched Altmetric Explorer using search terms such as “COVID” or “COVID-19” or “Coronavirus” or “SARS-CoV-2” or “nCoV” and then selected the top 100 articles with the highest AASs. For each article identified, we extracted the following information: the overall AAS, publishing journal, journal impact factor (IF), date of publication, language, country of origin, document type, main topic, and accessibility. The top 100 articles most frequently were published in journals with high (>10.0) IF (*n* = 67), were published between March and July 2020 (*n* = 67), were written in English (*n* = 100), originated in the United States (*n* = 45), were original articles (*n* = 59), dealt with treatment and clinical manifestations (*n* = 33), and had open access (*n* = 98). Our study provides important information pertaining to the dissemination of scientific knowledge about COVID-19 in online media.

## 1. Introduction

The severe acute respiratory syndrome coronavirus 2 (SARS-CoV-2) or 2019 novel coronavirus (2019-nCoV), which caused coronavirus disease 2019 (COVID-19)—a new infectious disease—was first detected in December 2019 in Wuhan, China [1]. COVID-19 rapidly spread worldwide [2], resulting in a global pandemic, as announced on 11 March 2020, by the World Health Organization (WHO) [3], and the situation is still dynamic. As of 31 December 2020, more than 81.1 million persons have been infected by SARS-CoV-2 across 222 countries or territories, and more than 1.7 million have died worldwide [3].

With the spread of the pandemic, a remarkably high number of scientific articles on COVID-19 have been published. Therefore, evaluating the impact of an article is particularly important, especially for readers who lack the time to read all relevant papers. Since the development of citation indexing in the 1960s, the number of citations has been widely used to measure the impact of articles. However, citations take a long time to accumulate and fail to reflect the impact of an article outside the academic community [4,5].

With the recent advent of social media, the concept of “altmetrics”—referring to non-traditional alternative article-level metrics—was introduced in the early 2010s. In comparison to traditional citation metrics, altmetrics measure the impact of an article after publication, based on its number of mentions across various online sources. This new metric can evaluate the instantaneous influence of published work on broad audiences in comparison to citation counts, thereby indicating the articles’ dissemination to the general public [6,7].

To date, several studies have been conducted to identify the top altmetric articles in specific medical fields [8,9,10,11,12]. However, to the best of our knowledge, no bibliometric analysis of top altmetric articles related to COVID-19 have been reported. Therefore, this study’s aim was to identify the top 100 COVID-19 related altmetric scientific publications and analyze their characteristics.

## 2. Materials and Methods

The present study did not involve human subjects and thus did not require approval from the institutional review board.

### 2.1. Selection of Articles

We conducted a search for scientific publications on COVID-19 using “Advanced Search” in Altmetric Explorer (Altmetric LLP, London, UK). To identify the closest matching publication, the search terms applied included “COVID” or “COVID-19” or “Coronavirus” or “SARS-CoV-2” or “nCoV”, which were used as keywords in the title. No restrictions were applied regarding journal, language, date of publication, output title, output type, or scholarly identifiers.

Altmetric.com, one of the main providers of alternative indicators, was chosen for this study because it is the most comprehensive source covering the vast majority of online media activities associated with scientific papers. We obtained permission to access the Altmetric database, which contains almost 31 million research articles from more than 36,000 journals and captures real-time mentions in public policy documents, blogs, mainstream media, online reference managers, such as Mendeley, research highlights, post-publication peer review platforms, Open Syllabus, YouTube, and social media networks, including Facebook and Twitter. Altmetric.com provides Altmetric Attention Scores (AASs) to measure the overall level of online impact arising from a particular research output, which is presented as a whole number. The AAS is a weighted score of total mentions of an article across various online media, reflecting the anticipated relative degrees of influence of sources on potential readers (e.g., default weights of: 8 for news outlets, 5 for science blogs, 3 for Wikipedia or policy documents, 1 for Twitter, and 0.25 for Facebook) [7].

The AASs of all articles were recorded and then compiled into a single database. Then, these articles were ranked in descending order, based on their AASs for selecting the top 100 articles with the highest AAS. To determine if the articles were mainly focused on COVID-19, two authors (J.Y.M. and D.Y.Y.) independently reviewed each article’s title and abstract (and even the full text, if required) and generated the final lists of the top 100 articles with the highest AAS. There was no disagreement between the two reviewers in the selection of top 100 articles on COVID-19. Both reviewers excluded three articles among 103 articles with the highest AAS because two original articles mainly focused on other coronavirus family outbreaks rather than COVID-19, and one news report could not be discovered with the Internet search for the title on 29 December 2020.

### 2.2. Data Extraction from Articles

The abstract and full text for each article identified were independently reviewed by the same two authors, who extracted the following information: (1) overall AAS, (2) publishing journal, (3) its journal impact factor (IF) based on the Journal Citation Reports (JCR) 2019 edition (Clarivate Analytics, Philadelphia, Pennsylvania, USA), (4) month and year of publication, (5) language, (6) country of origin, (7) document type (original articles (clinical observational study, basic study, randomized controlled trial, systematic review/meta-analysis), reviews, case reports, letters, news, editorials, etc.), (8) main topics (virus, epidemiology, immunology, transmission, prevention, clinical manifestations, treatment, public health responses, vaccines, or miscellaneous (i.e., not conforming to one of the categories listed)); and (9) accessibility (open-access or pay-for-access).

The Altmetric Explorer search was conducted on a specific day (29 December 2020) to avoid changes in the scores of articles. In only two cases in which disagreement occurred between two reviewers regarding the main topic of articles, the articles were reviewed jointly and discussed with a third reviewer (E.J.Y.) until consensus was reached. The country of origin was defined by the address provided for the authors. If the authors were affiliated with more than one country, it was classified as an international collaboration.

### 2.3. Analysis

This study adopted descriptive statistics using counts and proportions to describe the articles included.

## 3. Results

The top 100 articles on COVID-19 with the highest AAS were listed in Supplementary File S1 attached. The AAS of the top 100 articles ranged from 7301–34,789. The article ranked number 1 was “The proximal origin of SARS-CoV-2” by Andersen et al. published in Nature Medicine in March 17, 2020 [13].

The top 100 articles were published in 35 journals, led by the New England Journal of Medicine (*n* = 17), followed by the Lancet (*n* = 11) (Table 1). If journals in each subject category were divided into four quartiles based on their IF, the proportion of Q1 journals (84.0%) was much higher than those of Q2 (2.0%), Q3 (2.0%), and Q4 (0%) journals and journals with no IF (12.0%).

**Figure 1:**
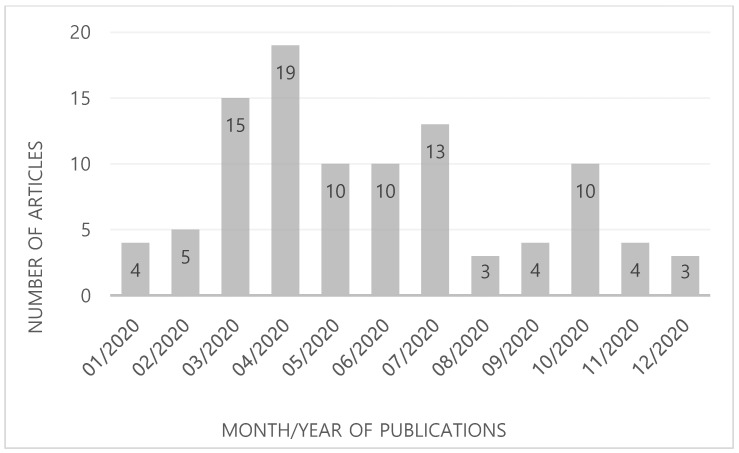
Publication month and year of the Top 100 articles about COVID-19 with the highest Altmetric Attention Scores.

A total of 67 articles were published in journals with an IF > 10.0, whereas 12 papers appeared in preprint online databases, such as MedRxiv and BioRxiv. All the articles were published in 2020, with 19 and 15 articles being published in April and March, respectively (Figure 1).

**Figure 2:**
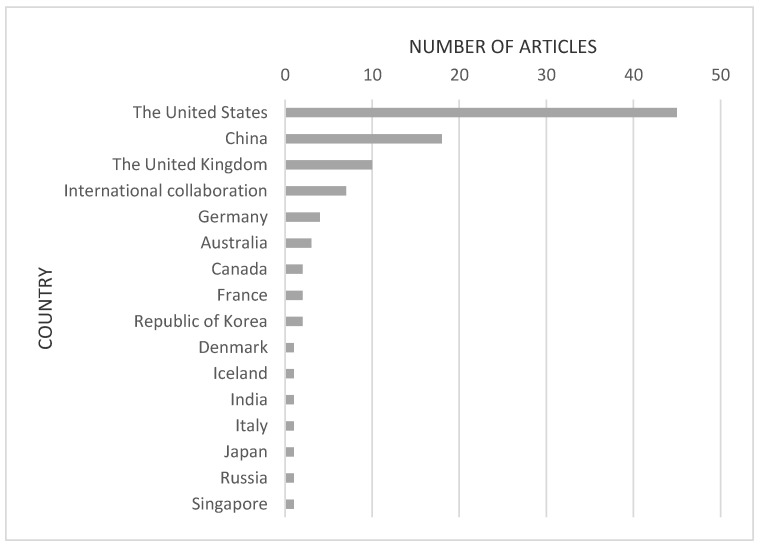
Countries of origin of the top 100 articles about COVID-19 with the highest Altmetric Attention Scores.

The United States published the most articles (*n* = 45), followed by China (*n* = 18), and the United Kingdom (*n* = 10). Seven were conducted through international collaborations (Figure 2).

As for the document type, original articles comprised the majority of the top 100 articles (*n* = 59), of which 28 were clinical observational studies. For the remaining articles, the most common document types were letters (*n* = 20) and case reports (*n* = 15). The most common topics were treatment (*n* = 18), clinical manifestations (*n* = 15), transmission (*n* = 14), epidemiology (*n* = 13), and virology (*n* = 11). All the publications were in English and 98 papers had open-access (Table 2).

## 4. Discussion

The development of altmetrics has been accompanied by an increase in the use of social media and a growth in tools for real-time impact of research. Altmetrics tools capture and aggregate information from various resources, such as mentions, clicks, views on the online media, Wikipedia, downloads, and many other digital interaction parameters [6,7]. Many previous studies have examined the relationship between citations and altmetrics, and most have shown that although citations and altmetrics are to some extent positively correlated, their correlations are very weak [14,15]. This finding suggests that citations and altmetrics indicate different aspects of the impact of scientific publications. Altmetrics may be considered as potential measures of a “disseminative impact” on the general population, whereas traditional citation-based metrics are statistics of “scholarly impact” on an academic-based audience [7].

In this study, we observed that overall, the altmetric top 100 COVID-19 research articles received remarkably high attention on social media and other online platforms, as compared with other biomedical fields. Previous studies reported that the top ranked articles had AASs between 176 and 3873 [8,9,10,11,12]; whereas in our study, the articles that ranked first, fiftieth, and hundredth had AASs of 34,789, 10,643, and 7301, respectively. This finding reflects the general public’s great interest in COVID-19.

We found that 67 articles of the altmetric top 100 articles were published in journals with an IF > 10.0 according to the JCR 2019 edition. This finding highlights that journals with a high IF attract a wider readership, spanning specialized professionals to general audiences. It is not surprising that the articles appearing in prestigious journals with high IFs are more frequently referenced in news, as well as social media and other databases, as compared to papers published in more obscure journals. Our study also found that the altmetric top 100 articles on COVID-19 were published more frequently during the early pandemic period (between March and July 2020). A total of 67 articles were published during the early pandemic, whereas 24 were published later in the pandemic (between August and December 2020). This could be attributed to the public’s greater interest and need to receive information regarding the novel infectious disease during the pandemic’s early phase.

In our study, the United States was the country with the most articles in the top 100, distantly followed by China. The dominance of the United States in the altmetric top 100 articles is in line with previous studies in other fields [8,9,10,11,12], which can be attributed to its large population, massive scientific output, and the large size of its online community. In addition, as COVID-19 was first reported in Wuhan, a fairly large number of important articles by Chinese scholars were published in early 2020.

With regard to the document type of articles, although original articles accounted for 59 of the altmetric top 100, there were various document types such as original articles, letters, case reports, editorials, as well as news in our list of the altmetric top 100 COVID-19 articles. This reflects the public’s diverse and broad interests in COVID-19. In particular, scientific publications providing information that is newer and easier to understand are the ones that attract the greater audiences in online media. This is in contrast to the results of the majority of classic citation studies, which reported that the most top-cited articles were typically original articles and reviews [16,17]. Although public interests encompass many diverse topics, the ones that appeared most frequently in the top altmetric articles were treatment and clinical manifestations of COVID-19, which highlight the importance of clinical management among the public, and reflect their fear and anxiety about the global spread of the unknown virus. Finally, our study found that 98 of the top 100 articles were open-access. Open accessibility can serve as an important component of achieving high altmetric scores because open-access articles provide wider public access than pay-for-access articles.

In the case of the COVID-19 outbreak, social media could be an increasingly important part of public health [18]. People use social media not only for showing personal emotions and opinions but also for seeking information regarding the outbreak. Social media plays a critical role in transmitting reliable information from national health authorities, public health experts, and scientists to the general public.

However, during the COVID-19 pandemic, social media also had a dark side―rumors, stigma, conspiracy theories, etc. Such misinformation can have potentially serious implications on individuals and communities in dealing with the pandemic crisis [19]. Unfortunately, another problem that cannot be neglected is the quality of the research that is being done and published during the COVID-19 pandemic. During the pandemic, scientific publication has become complicated because of the exponential growth of manuscripts related to COVID-19, shortage of experts available for peer review, and the need for rapid publication [20].Predictably, these problems in the current publication environment have led to the large number of corrections and retractions of published papers [21,22]. The publication of pervasive incorrect data, whether as a result of honest error or misconduct, may result in a major change in the direction of future studies and clinical decision making that can affect patient care.

In addition, there are several public key players, who play a pivotal role with regard to COVID-19 information in social networks. For example, the number 4 ranked article, “Hydroxychloroquine or chloroquine with or without a macrolide for treatment of COVID-19: A multinational registry analysis” was published in the 22 May 2020, issue of the Lancet [23]. The news about the use of chloroquine for the prevention or treatment of COVID-19 was widespread during the early phase of the COVID-19 pandemic, despite warnings that it had not been proven to be of any help against the coronavirus [24]. On 18 May 2020, the United States president Donald Trump said that he had been taking an anti-malaria drug hydroxychloroquine daily for over a week to prevent the coronavirus infection. President Trump has been a key player in social networks, which implies that for solving the COVID-19 problem, people’s interests are heavily concentrated on his behavior [18]. Hence, his comments and subsequent debates on social networks may have led to the people’s explosive interest. However, the aforementioned article was retracted in June 2020 because there were doubts on the veracity of its primary data sources. Interestingly, the number 5 ranked article was the retraction notice of the number 4 ranked article [25].

Despite these negative aspects, social media represents a uniquely powerful tool for the widespread dissemination of scholarly publications Thus, our list of the altmetric top 100 COVID-19 articles provides an insight into the dissemination of scientific knowledge through online media during a defined short period of time, following the emergence of the novel infectious disease.

Our study has some limitations. First, for assessing alternative metrics, we solely used data supplied by Altmetric.com. We selected it because of its wide use and inclusion of a comprehensive range of online platforms. Our results might have been slightly different if we had used other altmetric tools that aggregate and provide article-level metrics, such as PlumX, ImpactStory, and PLoS Impact Explorer [26]. Second, we used specific search terms to delineate scientific publications related to COVID-19. Thus, some potential articles may have been missed because of their using other words or terms. Finally, there are certain inherent shortcomings that should be considered in the use of altmetrics that do not cover the demographics of scientists or lay people interacting with online research material and the nature of each mention (positive or negative, simple mention, or in-depth discussion). In addition, the credibility of commentators and the validity of their comments are uncertain due to the fact that online data are much easier to manipulate [27,28,29].

In conclusion, our study presents a detailed list and analysis of the altmetric top 100 articles on COVID-19 research, thereby providing important information pertaining to the dissemination of scientific knowledge during the pandemic caused by a novel virus.

## Figures and Tables

**Table 1:** Journals that published two or more of the top 100 articles about coronavirus disease 2019 (COVID-19) with the highest Altmetric Attention Scores.

Journal	Impact Factor *	No. of Articles
New England Journal of Medicine	74.699	17
Lancet	60.390	11
MMWR: Morbidity and Mortality Weekly Report	13.606	8
MedRxiv	N/A	6
Science	41.846	6
Emerging Infectious Diseases	6.259	5
JAMA: Journal of the American Medical Association	45.540	5
Annals of Internal Medicine	21.317	4
Nature	42.779	4
BioRxiv	N/A	3
Nature Medicine	36.130	3
British Medical Journal	30.313	2
International Journal of Antimicrobial Agents	4.621	2
Lancet Infectious Diseases	24.446	2
Proceedings of the National Academy of Sciences of the United States of America	9.412	2

* Calculated using Journal Citation Reports for the year 2019.

**Table 2:** Document types, main topics, and accessibility of the top 100 articles about COVID-19 with the highest Altmetric Attention Scores.

Document Type	No. of Articles
Original article	59
Clinical observational studies	28
Basic studies	15
Randomized controlled trials	9
Systematic review/Meta-analysis	7
Letters	20
Case reports	15
Editorials	3
News	2
Retraction notice	1
Main topic	No. of Articles
Treatment	18
Clinical manifestations	15
Transmission	14
Epidemiology	13
Virology	11
Prevention	8
Public health response	5
Vaccines	5
Immunology	4
Miscellaneous	7
Accessibility	No. of Articles
Open-access	98
Pay-for-access	2

## Data Availability

Data cannot be shared because there are embargoes on datasets. Anonymized data will be shared by request from any qualified investigators.

## Supplementary Materials

The following are available online at https://www.mdpi.com/2227-9032/9/2/239/s1, Supplementary File S1.
